# New evidence on the validity of the selection methods for recruitment to general practice training: a cohort study

**DOI:** 10.3399/BJGPO.2023.0167

**Published:** 2024-05-15

**Authors:** Paul A Tiffin, Emma Morley, Lewis W Paton, Fiona Patterson

**Affiliations:** 1 Health Professions Education Unit, Hull York Medical School, University of York, York, UK; 2 Work Psychology Group, Derby, UK

**Keywords:** selection, training, predictive validity, general practice, primary health care, cohort studies

## Abstract

**Background:**

Selection into UK-based GP training has used the Multi-Specialty Recruitment Assessment (MSRA) and a face-to-face selection centre (SC). The MSRA comprises of a situational judgement test and clinical problem-solving test. The SC was suspended during the COVID-19 pandemic. Evidence is needed to guide national and international selection policy.

**Aim:**

To evaluate the validity of GP training selection.

**Design & setting:**

A retrospective cohort study using data from UK-based national recruitment to GP training, from 2015–2021.

**Method:**

Data were available for 32 215 GP training applicants. The ability of scores from the specialty selection process to predict subsequent performance in the Clinical Skills Assessment (CSA) of the Membership of the Royal College of General Practitioners examination was modelled using path analysis. The effect sizes for sex, professional family background, and world region of qualification were estimated.

**Results:**

All component scores of the selection process demonstrated statistically significant independent relationships with CSA performance (*P*<0.001), thus establishing their predictive validity. All were sensitive to demographic factors. The SC scores had the weakest relationship with future CSA performance. However, for candidates with MSRA scores below the lowest quartile, the relative contribution of the SC scores to predicting CSA performance was similar to that observed for MSRA components.

**Conclusion:**

The MSRA has predictive validity in this context. Re-instituting an SC for those with relatively low MSRA scores should be considered. However, the relative costs and potential advantages and disadvantages should be carefully weighed.

## How this fits in

The added value of a face-to-face selection centre (SC) for recruitment into GP training has been questioned. We found that performance on all components of GP training selection was independently related to eventual performance in the Clinical Skills Assessment (CSA). However, the relationship with the SC scores was relatively weak. Re-instituting SCs is unlikely to add substantial value in this context, although it could be considered for candidates with relatively low scores on the Multi-Specialty Recruitment Assessment (MSRA).

## Introduction

There are well-recognised, long-term workforce shortages in GPs both in the UK and internationally.^1,2^ The selection methods used to recruit applicants into GP training should accurately identify those doctors likely to complete training and sustainably work effectively in primary care. Previous systematic reviews of international selection practices demonstrate the need to provide evidence of reliability, validity, and fairness.^3,4^ There have been several previous studies evaluating data relating to the UK GP selection process^5,6^ and this article presents new, large-scale evidence.

Since 2006, selection into UK GP training has been standardised and centralised through a national recruitment office.^7^ Before the COVID-19 pandemic the process comprised of the following three stages:

- Stage 1: administrative, in which proof of eligibility and educational qualifications are checked.
- Stage 2: the MSRA is taken.
- Stage 3: SC, in which there is a face-to-face clinical examination and written test.

The MSRA consists of a situational judgement test (SJT; known as the professional dilemmas paper) and a clinical knowledge test (the Clinical Problem-Solving [CPS] paper). The MSRA aims to assess the clinical and interpersonal knowledge expected in a doctor completing their UK foundation years (FY) training. Training offers were determined by candidates’ national ranking on their combined MSRA and SC scores. From 2016–2020 doctors scoring above an MSRA threshold (the ‘bypass score’) were exempt from the SC assessment. This score was 575 points, except in 2020, when it was 550. During the COVID-19 pandemic the SC stage was suspended and offer decisions were informed solely by the MSRA scores.

Once selected, doctors enter a 3-year programme of hospital and primary care posts. During training they must pass the Membership examination of the Royal College of General Practitioners (MRCGP). This includes written components (the Applied Knowledge Test; AKT) and Clinical Skills Assessment (CSA). Further details are included in Supplementary Information S1. Previously, it has been shown that performance on the MSRA validly predicts future achievement on the MRCGP.^8–10^ Two separate studies also raised issues regarding the value of the SC,^5,6^ since the scores accounted for only about 3% or 4% of additional variance once adjusted for MSRA performance. However, these studies treated the MSRA components as confounders rather than mediators of the relationship between SC and CSA performance. This introduced the risk of the so called ‘table 2 fallacy’.^11^ The authors also highlighted that the alternate forms reliability of the SC averaged only about 0.50 and also highlighted that abolishing the stage could save around £3 million over 3 years.^6^

Building on these prior investigations, the overall aims of this study were:

1. to evaluate the incremental validity (that is, the ability to predict the outcome, independent of the other measures) of the selection assessments in all applicants and in those with low MSRA scores; and
2. to evaluate the impact of key demographic characteristics (for example, sex and ethnicity).

The findings would guide policy on general practice and other specialty selection, both in the UK and elsewhere. For example, similar approaches are currently used for postgraduate selection in Australasia.^4^

## Method

### Ethics

The use of data from the UK Medical Education Database (UKMED) is not reliant on individual consent. However, any findings published from the UKMED must be presented in blunted form.^12^ Thus, all frequencies are rounded to the nearest multiple of five.

### Data processing and management

**Figure 1.**
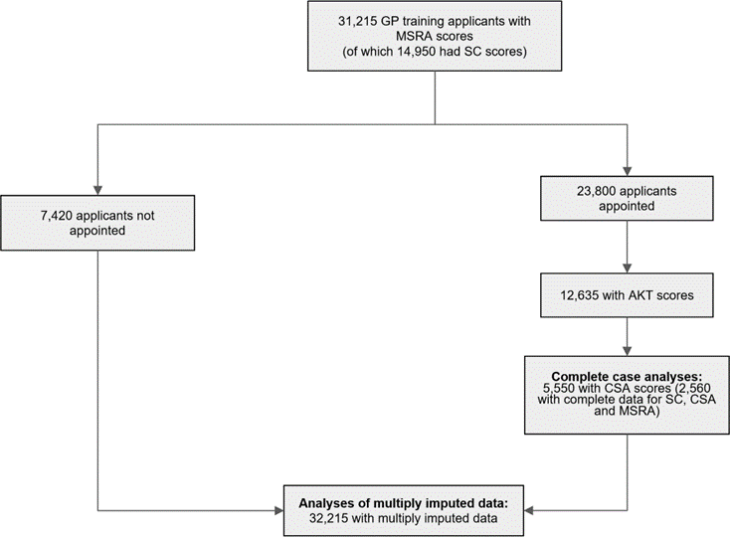
Flow of data through the study. Note that owing to blunting the numbers may not sum to the exact values. AKT = Applied Knowledge Test. CSA = Clinical Skills Assessment. MSRA = Multi-Specialty Recruitment Assessment. SC = selection centre.

Data were available, via the UKMED, for 32 215 applicants to the GP training scheme during the period 2015–2021. The flow of study data is shown in Figure 1.

### Selection measures

The SJT comprises of 50 workplace-based scenarios linked to interpersonally oriented content domains including ‘professional integrity’, ‘coping with pressure’, and ‘empathy and sensitivity’. Items use either a ranking response format or a multiple-choice format. The CPS paper has 97 questions in single best answer or enhanced matching format, assessing medical knowledge, presenting clinical scenarios for context. The scores are standardised across cohorts and summed to provide an overall MSRA score.

The SC is a set of face-to-face assessments, with three simulated consultation stations with actors and a written exercise. The latter was a short essay involving prioritising tasks in a clinical scenario.

To address the equivalence of scores on the MRCGP components over time, the marks ‘relative to the pass’ were used. ‘Low MSRA’ scores were classed as those below the bottom quartile for the study sample (466 marks). An explanation of why this definition was selected is provided in the Supplementary Information S4.

### Missing data handling

The outcome (CSA score) was only observed in those appointed to the scheme and sitting the exam within the study timeframe. This ‘range restriction’ in personnel selection studies is a special case of missing data.^13^ Consequently, missing values for both predictors and outcomes were imputed using chained equations. This is a valid approach to addressing this issue.^14–16^ As a sensitivity analysis, imputed and non-imputed results were compared. Further details on the handling of missing data are available in the Supplementary Information S3.

### Analysis approach

Univariable analyses were conducted. The potential impact of various educational and demographic characteristics (ethnicity, sex, and place of qualification) on the elements of the MSRA and the SC score were estimated via effect sizes. Either Cohen’s *d* or Glass’ Delta were calculated depending on equality of the intra-group variances. As some of the SC scores were imputed (owing to ‘bypass’ scores) the effect sizes were derived using the miesize package.^17^ Ethnicity was self-reported and dichotomised into those identifying as White and those identifying as Non-White for the purposes of analysis. There was almost complete overlap between ethnicity and place of primary medical qualification (PMQ). Thus, ethnicity was only analysed in this respect for UK graduates. Also, when building the multivariable model, only the selection assessment scores were entered, as these were relevant to the selection decision.

### Path analysis

Building multivariable models potentially gives rise to the 'table 2 fallacy'.^11^ This assumes that all the predictor variables entered into the model are confounders, rather than mediators, moderators, or colliders ('reverse confounders'). This can be addressed by using structural equation modelling to model the underlying causal relationship between the variables. Thus, a path model was developed informed by prior research^18,19^ (Supplementary Information S2 and Supplementary Figure S1). Stata/MP (version 17.0) and Mplus (version 8.8) were used to manage and analyse the data. All code is publicly available (https://github.com/pat512-star/P155).

## Results

Table 1 summarises the demographic details for applicants and entrants. The educational and academic performance for applicants are included in Supplementary Table S1. From this point, all the results shown are derived from the imputed data (*m* = 10) unless otherwise indicated.

**Table 1.** A breakdown of the demographic variables for those applicants to GP training with and without the primary outcome of interest (CSA score at first attempt)

Demographic variable	Applicants not entering scheme, *n* (%)	Entrants with at least one CSA attempt, *n* (%)	All applicants, *n* (%)	Missing values, *n* (%)
Male sex	3465/7420 (46.7)	2240/5550 (40.4)	12 800/31 215 (41.0)	0/31 215 (0.0)
Non-professional socioeconomic background	555/3145 (17.6)	685/3610 (19.0)	2995/15 490 (19.3)	15 725/31 215 (50.4)
BAME (UK graduates only)	1950/4485 (43.5)	1655/4440 (37.3)	7800/20 140 (38.7)	665/20 805 (3.2)
**Place of qualifications**				
UK	4655/7420 (62.7)	4585/5550 (82.6)	20 805/31 215 (66.7)	0/31 215 (0.0)
EEA	420/7420 (5.7)	190/5550 (3.4)	1520/31 215 (4.9)	0/31 215 (0.0)
IMG	2345/7420 (31.6)	775/5550 (14.0)	8895/31 215 (28.5)	0/31 215 (0.0)

BAME = Black, Asian and Minority Ethnic. CSA = Clinical Skills Assessment. EEA = European Economic Area. IMG = international medical graduate.

### Univariable results

#### Univariable relationship between the measures

The correlations (Spearman’s *P* values) between the key measures are shown in Supplementary Table S2. The results of the univariable analysis between the predictors and the CSA scores are shown in Table 2. As can be seen, all three selection measure scores are significantly associated with predictive of CSA performance (*P*<0.001).

**Table 2.** Results from the univariable and multivariable linear regression analyses predicting CSA performance from the scores from the three selection measures (CPS, SJT, and SC) on the multiply imputed study data (*m* = 10) for the whole sample and for different subgroups of applicants. The last three rows also report the results from analysis of the non-imputed data

Selection assessment	Coefficient (β)	*P* value	Lower 95% CI	Upper 95% CI	*R* ^2^ for the model^a^
**Univariable results**					
Clinical problem solving	0.17 (0.51)	<0.001	0.16	0.17	0.26
Situational judgement test	0.18 (0.56)	<0.001	0.17	0.19	0.31
Selection centre	0.91 (0.39)	<0.001	0.83	0.98	0.15
**Multivariable results**					
All applicants					
Clinical problem solving	0.09 (0.28)	<0.001	0.08	0.10	0.40
Situational judgement test	0.11 (0.34)	<0.001	0.10	0.12	
Selection centre	0.43 (0.18)	<0.001	0.33	0.53	
Applicants scoring below the first quartile on the MSRA					
Clinical problem solving	0.06 (0.15)	<0.001	0.05	0.07	0.12
Situational judgement test	0.08 (0.21)	<0.001	0.07	0.10	
Selection centre	0.42 (0.21)	<0.001	0.32	0.51	
Results from non-imputed data					
Clinical problem solving	0.10 (0.27)	<0.001	0.09	0.11	0.30
Situational judgement test	0.12 (0.32)	<0.001	0.10	0.13	
Selection centre	0.47 (0.17)	<0.001	0.37	0.56	

^a^This is the mean *R* ^2^ for the models derived from the imputed data. CPS = clinical problem-solving. CSA = Clinical Skills Assessment. SC = selection centre. SJT = situational judgement test.

### Sensitivity to demographic characteristics

**Figure 2.**
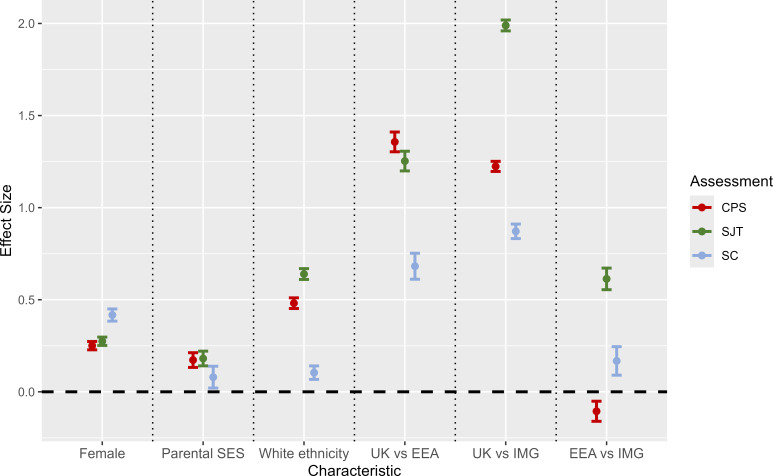
The effect size of key demographic characteristics on performance at the three selection assessments (clinical problem-solving [CPS] test, situational judgement test [SJT], and selection centre [SC]). EEA = European Economic Area. IMG = international medical graduate. SES = socioeconomic status according to occupational categorisation ('professional' versus 'non-professional').

The effect sizes for the three selection measures are shown in Figure 2 (see also Supplementary Table S3). The largest effect sizes are observed for place of PMQ, with UK graduates scoring higher, on average, on all measures, compared with non-UK graduates. The SC scores are those most sensitive to sex (*d* = 0.42). However, the SC scores are less sensitive to socioeconomic status than the MSRA scores, although the confidence intervals overlap slightly in this respect. In contrast to the MSRA components, only a modest impact of ethnicity is observed for the SC scores. Note that the effect of ethnicity was only estimated in UK graduates.

### Multivariable results

The multivariable results for the prediction of CSA performance from CPS, SJT, and SC are shown in Table 2. Once the influence of SJT and CPS performance is controlled for, the SC ratings have only modest independent predictive ability in relation to the CSA score. Note, the overall predictive power of the SJT and CPS scores are weakened for those applicants with relatively low MSRA scores.

### Path analysis results

**Figure 3.**
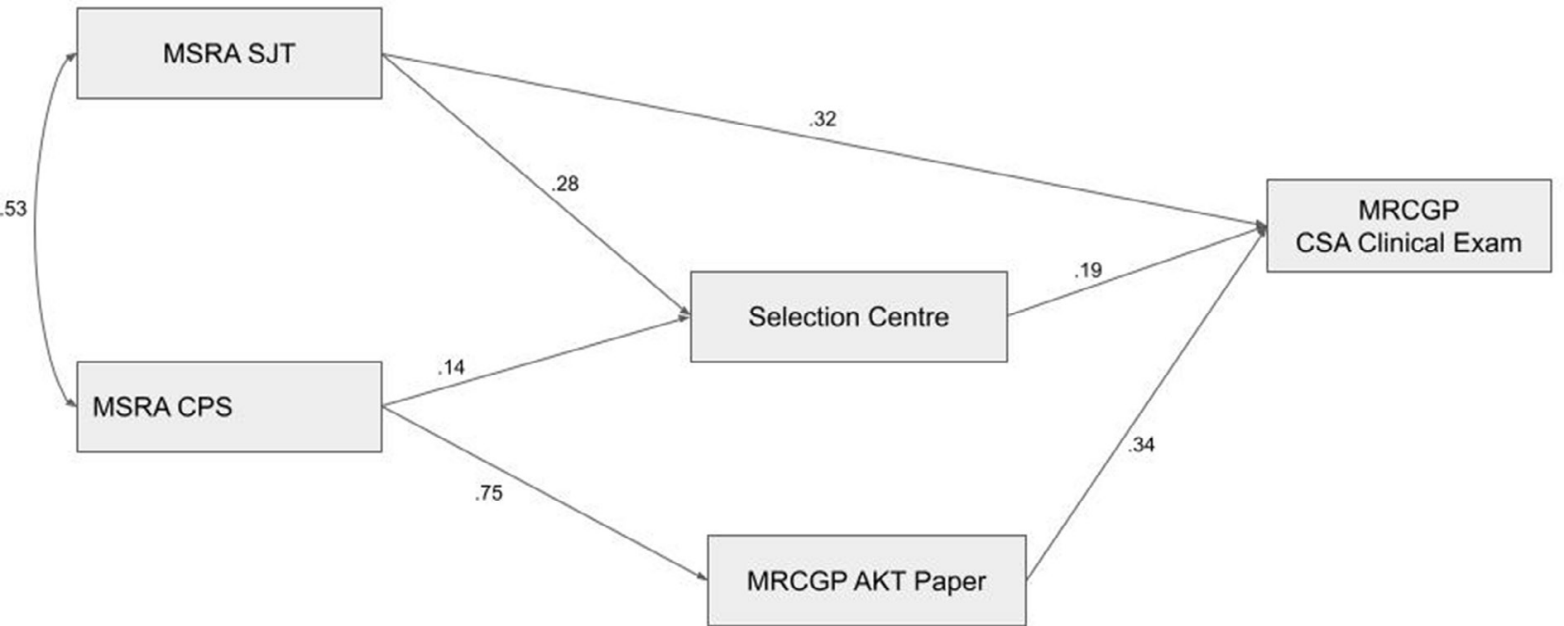
The a priori theoretical model for GP selection, testing the relationship between the predictors and outcome (CSA score) in the multiply imputed study data (*m* = 10) for all applicants (*n* = 31 215). AKT = Applied Knowledge Test. CPS = clinical problem-solving. CSA = Clinical Skills Assessment. MSRA = Multi-Specialty Recruitment Assessment. SJT = situational judgement test.

**Figure 4.**
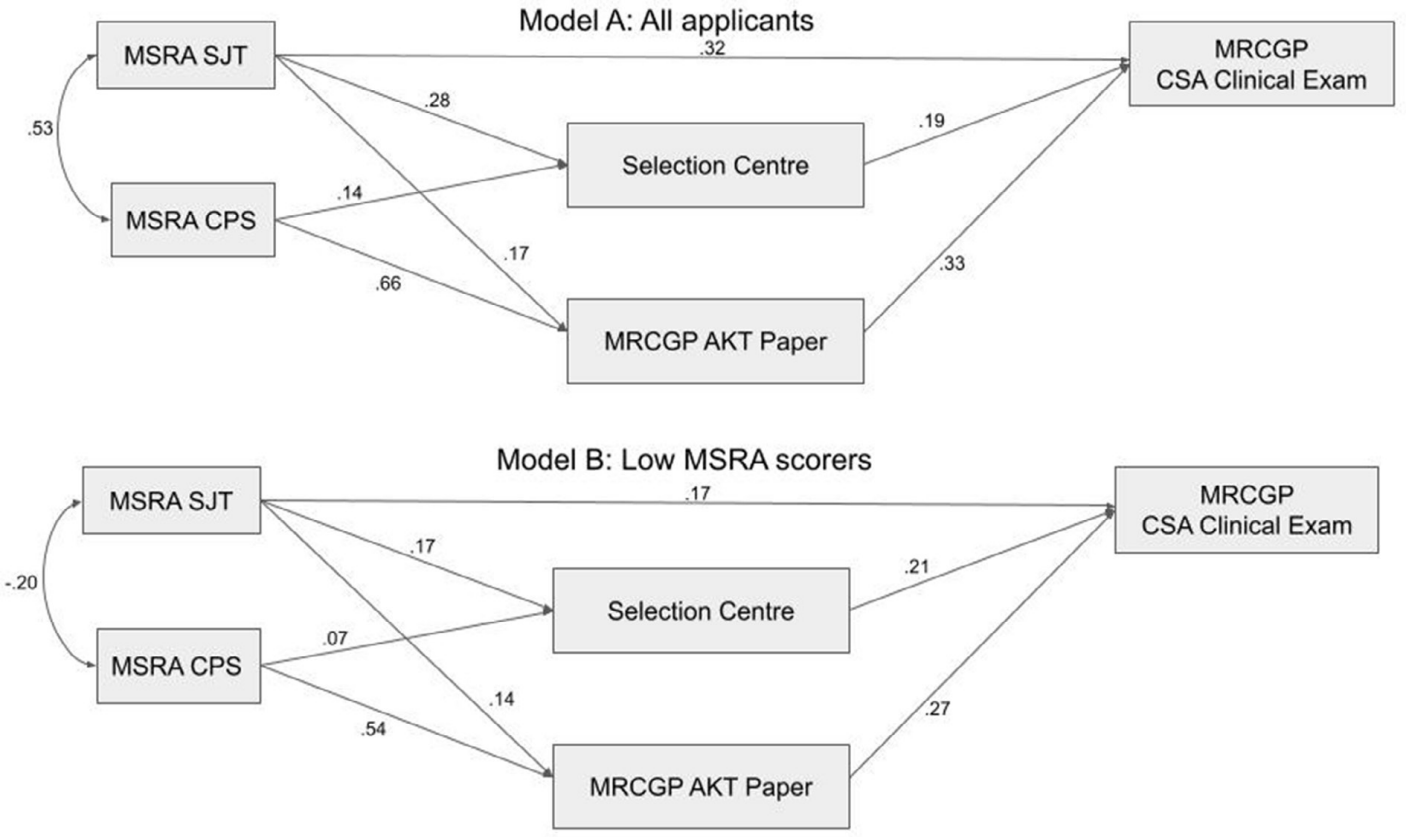
Path models modified according to modification indices, testing the relationship between the predictors and outcome (CSA score) in the multiply imputed study data (*m* = 10) for all applicants (Model A, *n* = 31 215) and for those scoring below the lowest quartile on the MSRA (Model B, *n* = 7795). AKT = Applied Knowledge Test. CPS = clinical problem-solving. CSA = Clinical Skills Assessment. MSRA = Multi-Specialty Recruitment Assessment. SJT = situational judgement test.

A path analysis, based on the a priori hypothesised model, was estimated in the imputed datasets (*m* = 10). The initial model showed close to an acceptable fit to the data, according to the Comparative Fit Index (CFI) at 0.96. However, the Tucker–Lewis Index (TLI) was only around 0.89, where 0.90 is considered to indicate acceptable fit (see Figure 3, Supplementary Figure S2, and Supplementary Table S4). Modification indices suggested considerable improvement in fit could be achieved by allowing the AKT score to be regressed on the SJT score. This suggested that the procedural knowledge tested by the SJT was also relevant to answering the AKT questions, and/or that performance on both assessments was at least partly determined by a shared ability. The resulting path model (Figure 4A) showed a good fit to the data, with the mean CFI being nearly 1.00 (0.995) and the TLI being 0.98. The fit indices for the models are shown in Supplementary Table S4. The path model for those with relatively low MSRA scores is also shown in Figure 4B. The main difference to model A is the relatively reduced ability of the SJT scores to predict CSA performance. In contrast, the unique ability of the SC scores to predict CSA performance is consistent across models (β = 0.2). This infers around 4% of variance in the CSA scores is uniquely explained by the SC scores.

Results from the non-imputed data are shown in Supplementary Figures S3 and S4.

## Discussion

### Summary

In line with previous findings, we demonstrated that the MSRA scores predicted CSA performance. Also in line with prior findings, we observed the SC scores only incrementally predict an additional 3%–4% of variance in the CSA.^6^ However, we also showed that, for applicants with low MSRA scores, this is also true for the SJT and CPS scores.

Our findings add to the GP-selection evidence in several important ways. First, our path analyses produced unbiased estimates of the unique contribution of each predictor. Second, we showed the relative reduced sensitivity of the SC scores, compared with the MSRA assessments, to key demographic characteristics. These have implications for equality and diversity in recruitment. Third, we estimated our models in applicants with relatively low MSRA scores to evaluate the relative value of SCs in this group of candidates.

### Strengths and limitations

This was a relatively complete, national cohort of applicants. Nevertheless, there was the obvious challenge of not being able to observe the outcome of interest in those who had not been appointed or undertaken the CSA within the study timeframe. However, this issue was addressed using multiple imputation. Our imputed and non-imputed results did not meaningfully differ, providing evidence for the validity of this approach. Ideally our validity-related outcome would have been aspects of actual workplace behaviour. However, as these were not available, high-fidelity clinical simulation examinations may be the best available proxy for this. In this respect, US-based research evidence suggests that physician performance in postgraduate cardiology examinations predicts clinical outcomes in their patients experiencing myocardial infarction.^20^

### Comparison with existing literature

These results also have important general implications for the design of selection processes within postgraduate medicine and add to the international evidence base examining the validity of differing approaches.^4^ These relate to how well the constructs evaluated by selection assessments map to the validity criterion chosen. For example, in contrast, a similar evaluation of selection into psychiatry training noted that SC scores were moderately independently correlated with performance in the clinical membership examination.^19^ This may be because the SC used in psychiatry more closely resembles the subsequent clinical examination. Certainly, our findings are in keeping with the existing evidence for the use of selection approaches in postgraduate medical selection.^4^ That is, there is relatively strong evidence for the validity of scores derived from SJTs, CPS tests, and multiple mini-interviews. There is less consistent evidence for the validity of using curriculum vitae, references, and personal statements.

The sensitivity of the differing selection components to demographic factors have implications for equality and diversity. Specifically, in this context, the SJT was more sensitive to ethnicity and PMQ region than the other measures. Depending on their characteristics, such as the complexity of language or contextualisation of content, selection SJTs can be differentially sensitive to demographic factors.^21^ Thus, this issue is worth further researching. Differential attainment in clinical educational test scores between UK and non-UK medical graduates has been observed across numerous medical specialties.^22^ The drivers behind such phenomena are acknowledged to be complex and may include factors such as language fluency and cultural factors.^18,23^ In contrast, SC performance was less sensitive to ethnicity than the MSRA in this context. However, the relative insensitivity of the SC scores to demographics may be, at least partly, an artefact of its relatively low reliability.

### Implications for research and practice

Our findings support the continued use of the MSRA in this context. Moreover, they suggest that the re-introduction of some form of SC should be reconsidered for a smaller number of ‘borderline’ applicants, with relatively low scores on the MSRA. It is also possible that SCs could be made more reliable; for example, by reconfiguring the time allocated to create an increased number of shorter stations that could be delivered online, which would also significantly reduce costs.^24^ With the exception of sex, the SC scores seemed less sensitive to demographic factors, including ethnicity and world region of qualification. This implies placing some weight on an SC score, compared with the MSRA performance, could widen access to GP training. This should be considered and further explored. It may also be that candidates who perform poorly at SCs may benefit from early additional support and subsequently succeed at postgraduate training.^25^

The shortage of GPs in the workforce also needs to be considered. A previous study simulating changes to the GP selection system highlighted that completely removing MSRA cut-off scores would increase the number of trainees more than changing the selection process in other ways. However, it would also likely significantly increase the number of doctors failing to complete their GP training.^26^ There may also be patient safety and care-quality issues if selection procedures are not sufficiently robust. Thus, the issue of an MSRA cut-off (‘bypass’) score is complex. Further research could investigate how selection processes relate to other important outcomes, such as retention in the primary care workforce. Further modelling, taking a ‘Pareto-optimal’ perspective,^27^ could also be helpful in locating the optimum trade-off between educational performance and numbers of GP trainees recruited. Ideally, future evaluations should include cost-benefit analyses. These will inevitably be complex. They will have to account for both relatively direct costs (for example, temporary staff for vacant posts) and indirect costs (for example, those related to the risk of complaints and compensation relating to poor clinical practice). Such economic modelling will also take place in the context of a shifting workforce landscape with unpredictable trends relating to staff retention and medical migration, both to and from the UK.

The selection process is valid, in that performance on each component independently predicts future CSA performance. The MSRA scores are more predictive than those for the SC for CSA performance, but not for those with low MSRA scores. Thus, the use of an online SC for a relatively small number of 'borderline' candidates should be considered. This could address diversity issues and widening access to UK GP training. It may also optimise the absolute numbers of GP trainees. However, the potential costs and disadvantages should be weighed when making this decision.
